# Di-μ-iodido-bis­[acet­yl(4-methyl-2,6,7-trioxa-1-phosphabicyclo­[2.2.2]octa­ne)(*N*-nitroso-*N*-oxidoaniline-κ^2^ *O*,*O*′)rhodium(III)]

**DOI:** 10.1107/S1600536809043050

**Published:** 2009-11-07

**Authors:** Johan A. Venter, W. Purcell, H. G. Visser

**Affiliations:** a Department of Chemistry, University of the Free State, PO Box 339, Bloemfontein 9300, South Africa

## Abstract

The title compound, [Rh_2_(C_6_H_5_N_2_O_2_)_2_(C_2_H_3_O)_2_I_2_(C_5_H_9_O_3_P)_2_], contains a binuclear centrosymmetric Rh^III^ dimer bridged by iodide anions, with respective Rh⋯Rh and I⋯I distances of 4.1437 (5) and 3.9144 (5) Å. The Rh^III^ atom is in a distorted octa­hedral RhCI_2_O_2_P coordination with considerably different Rh—I distances to the bridging iodide anions. There are no classical hydrogen-bonding inter­actions observed for this complex.

## Related literature

For the synthesis of similar Rh complexes and information on oxidative addition products, see: Basson *et al.* (1984 ▶; 1986*a* ▶,*b* ▶; 1987 ▶, 1992 ▶); Roodt & Steyn (2000 ▶); Smit *et al.* (1994 ▶); Steyn *et al.* (1992 ▶); Van Leewen & Roobeeck (1981 ▶).
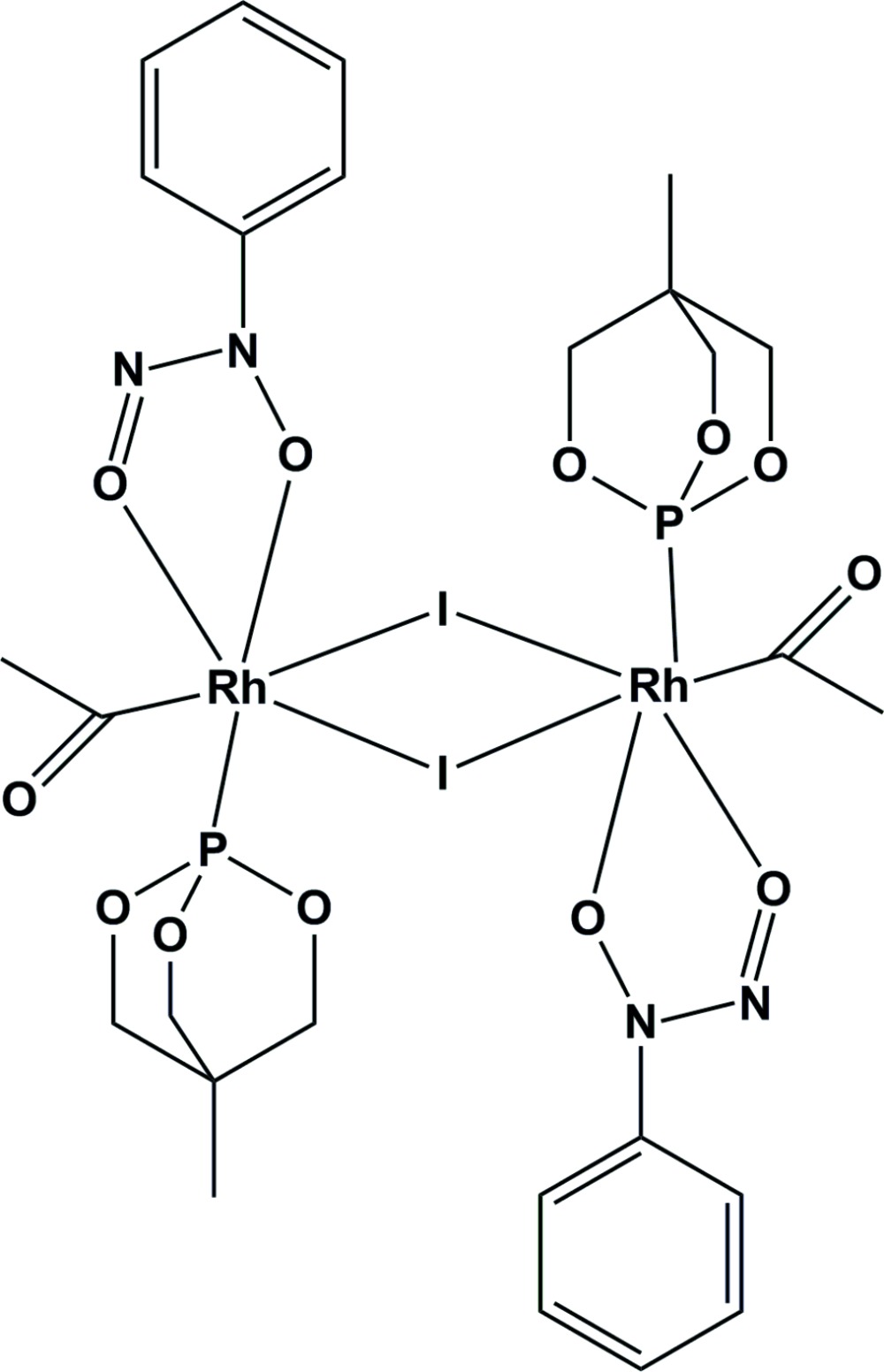


## Experimental

### 

#### Crystal data

[Rh_2_(C_6_H_5_N_2_O_2_)_2_(C_2_H_3_O)_2_I_2_(C_5_H_9_O_3_P)_2_]
*M* *_r_* = 1116.14
Monoclinic, 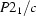

*a* = 10.055 (2) Å
*b* = 16.944 (3) Å
*c* = 11.149 (2) Å
β = 112.75 (3)°
*V* = 1751.7 (7) Å^3^
*Z* = 2
Mo *K*α radiation
μ = 2.86 mm^−1^
*T* = 293 K
0.10 × 0.08 × 0.06 mm

#### Data collection

Bruker SMART CCD 1K diffractometer
Absorption correction: multi-scan (*SADABS*; Bruker, 2004 ▶) *T*_min_ = 0.763, *T*_max_ = 0.847
12035 measured reflections
4344 independent reflections
3129 reflections with *I* > 2σ(*I*)
*R*_int_ = 0.051

#### Refinement

*R*[*F* ^2^ > 2σ(*F* ^2^)] = 0.033
*wR*(*F* ^2^) = 0.075
*S* = 0.95
4344 reflections
220 parameters
1 restraint
H-atom parameters constrained
Δρ_max_ = 0.93 e Å^−3^
Δρ_min_ = −0.50 e Å^−3^

### 

Data collection: *SMART* (Bruker, 2004 ▶); cell refinement: *SAINT-Plus* (Bruker, 2004 ▶); data reduction: *SAINT-Plus*; program(s) used to solve structure: *SIR97* (Altomare *et al.*, 1999 ▶); program(s) used to refine structure: *SHELXL97* (Sheldrick, 2008 ▶); molecular graphics: *DIAMOND* (Brandenburg & Putz, 2005 ▶); software used to prepare material for publication: *WinGX* (Farrugia, 1999 ▶).

## Supplementary Material

Crystal structure: contains datablocks global, I. DOI: 10.1107/S1600536809043050/wm2267sup1.cif

Structure factors: contains datablocks I. DOI: 10.1107/S1600536809043050/wm2267Isup2.hkl

Additional supplementary materials:  crystallographic information; 3D view; checkCIF report

## Figures and Tables

**Table:** 

C1—Rh	2.040 (8)
I—Rh	2.6351 (8)
Rh—O3	2.044 (5)
Rh—O2	2.052 (5)
Rh—P	2.186 (2)
Rh—I^i^	3.0511 (9)

**Table:** 

Rh—I—Rh^i^	93.30 (2)
C1—Rh—O3	92.9 (3)
C1—Rh—O2	92.2 (3)
O3—Rh—O2	78.74 (19)
O2—Rh—P	172.54 (15)
C1—Rh—I	93.9 (2)
O3—Rh—I	168.12 (14)
C1—Rh—I^i^	172.7 (2)

Symmetry code: (i) 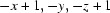.

**Fig. 1.**
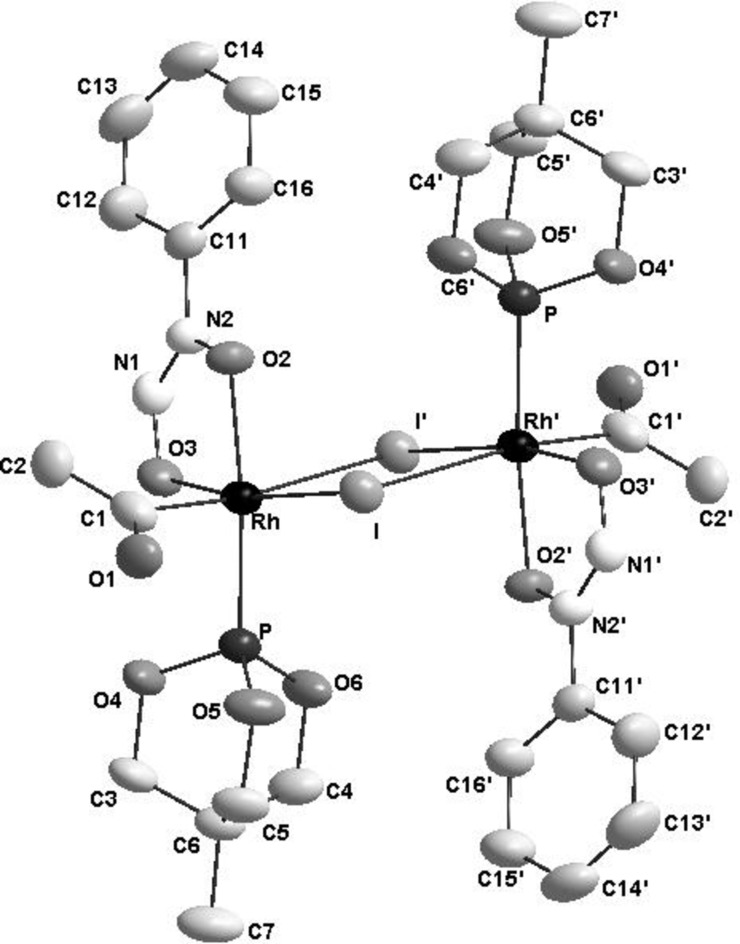
View of the dimeric compex present in the title compound. Probability level for displacement ellipsoids is 50%. Symmetry-related atoms are generated by the symmetry operator i) -x+1, -y, -z+1.

## supplementary crystallographic information

### Comment

The title compound (Fig. 1) is the product of the oxidative addition of CH_3_I to [Rh(cupf)(CO){P(OCH_2_)_3_CCH_3_}] (cupf = cupferrate, (C_6_H_5_N_2_O_2_) (Basson *et al.*, 1992) which forms part of a series of rhodium complexes used in the kinetic studies of these reactions (Basson *et al.*, 1984; 1986*b*; Steyn *et al.*, 1992; Smit *et al.*, 1994; Roodt & Steyn, 2000).

In the structure, the Rh^III^ metal centre is coordinated to two bridging iodide ligands, an acyl ligand, a cyclic phosphite ligand (P(OCH_2_)_3_CCH_3_) and an O,O'-bidentate cupferrate ligand. The coordination sphere around the metal is somewhat distorted from the octahedral geometry and a number of angles deviate significantly from the ideal (see Table 1). This is probably due to the small bite angle of 78.74 (19) ° formed by the cupferrate ligand and the metal centre. The angles, P—Rh—O2 of 172.54 (15) °, P—Rh—I of 96.21 (6)° and P—Rh—C1 of 87.2 (2) ° clearly support the visual impression of Fig. 1 showing the phosphite ligand bent outward to minimise steric interaction. The respective Rh—Rh and I—I distances were calculated as 4.1437 (5) and 3.9144 (5) Å. At 2.186 (2) Å the Rh—P bond lenght is short, compared to the 2.327 (4) Å of [Rh(cupf)(CO)(CH_3_)(I)(PPh_3_)] (Basson *et al.*, 1987). This stems from the nature of phosphites to be excellent π-acceptors, causing stronger back donation from rhodium resulting in a shorter Rh—P bond. Also, the sterically small cyclic phosphite ligand allows for a closer fit in the coordination sphere. The Rh-I' distance (symmetry operator -x+1, -y, -z+1), the one *trans* to the acyl ligand, is significantly longer than the other Rh—I distance, demonstrating the large *trans*-influence of the acyl ligand. The formation of [Rh(cupf)(COCH_3_)(µ-I){P(OCH_2_)_3_CCH_3_}]_2_ can most probably be attributed to the minor steric requirements of both the cupferrate ligand with its narrow bite angle and even more importantly the small cone angle of the phosphite. No classical hydrogen-bonding interactions are observed in the title compound.

### Experimental

The bicyclic phosphite ester, P(OCH_2_)_3_CCH_3_, and [Rh(C_6_H_5_N_2_O_2_)(CO)_2_] was prepared according to the respective methods reported previously (Van Leewen & Roobeeck, 1981; Basson et al., 1986a). Equimolar amounts of the cyclic phosphite was mixed with [Rh(C_6_H_5_N_2_O_2_)(CO)_2_] in acetone to form [Rh(C_6_H_5_N_2_O_2_)(CO)(P(OCH_2_)_3_CCH_3_)]. The reaction mixture was concentrated by evaporation after which a tenfold excess of CH_3_I was added. The container was covered with a perforated plastic film and left to stand for two days at 271 K after which brown-red single crystals of the title compound were isolated.

### Refinement

The methylene, aromatic and methyl H atoms were placed in geometrically idealized positions (C—H = 0.93 – 0.98 Å) and constrained to ride on their parent atoms with *U*_iso_(H) = 1.2*U*_eq_(C) for methylene and aromatic protons and *U*_iso_(H) = 1.5*U*_eq_(C) for methyl protons respectively. The highest residual electron density was located 0.93 Å from I.

### Crystal data

**Table:**

[Rh_2_(C_6_H_5_N_2_O_2_)_2_(C_2_H_3_O)_2_I_2_(C_5_H_9_O_3_P)_2_]	*F*(000) = 1080
*M**_r_* = 1116.14	*D*_x_ = 2.116 Mg m^−^^3^
Monoclinic, *P*2_1_/*c*	Mo *K*α radiation, λ = 0.71073 Å
Hall symbol: -P 2ybc	Cell parameters from 859 reflections
*a* = 10.055 (2) Å	θ = 2.3–28.1°
*b* = 16.944 (3) Å	µ = 2.86 mm^−^^1^
*c* = 11.149 (2) Å	*T* = 293 K
β = 112.75 (3)°	Cuboid, brown-red
*V* = 1751.7 (7) Å^3^	0.10 × 0.08 × 0.06 mm
*Z* = 2	

### Data collection

**Table:**

Bruker SMART CCD 1K diffractometer	4344 independent reflections
Radiation source: fine-focus sealed tube	3129 reflections with *I* > 2σ(*I*)
graphite	*R*_int_ = 0.051
ω scans	θ_max_ = 28.3°, θ_min_ = 2.2°
Absorption correction: multi-scan (*SADABS*; Bruker, 2004)	*h* = −13→11
*T*_min_ = 0.763, *T*_max_ = 0.847	*k* = −22→14
12035 measured reflections	*l* = −12→14

### Refinement

**Table:**

Refinement on *F*^2^	1 restraint
Least-squares matrix: full	H-atom parameters constrained
*R*[*F*^2^ > 2σ(*F*^2^)] = 0.033	*w* = 1/[σ^2^(*F*_o_^2^) + (0.0354*P*)^2^] where *P* = (*F*_o_^2^ + 2*F*_c_^2^)/3
*wR*(*F*^2^) = 0.075	(Δ/σ)_max_ < 0.001
*S* = 0.95	Δρ_max_ = 0.93 e Å^−^^3^
4344 reflections	Δρ_min_ = −0.49 e Å^−^^3^
220 parameters	

### Special details

**Table:**

Experimental. The intensity data were collected on a Bruker *SMART* CCD 1 K diffractometer using an exposure time of 20 s/frame. A total of 1315 frames were collected with a frame width of 0.3° covering up to θ = 28.29° with 99.8% completeness accomplished. The first 50 frames were recollected at the end of the data collection to check for decay; none was found.
Geometry. All e.s.d.'s (except the e.s.d. in the dihedral angle between two l.s. planes) are estimated using the full covariance matrix. The cell e.s.d.'s are taken into account individually in the estimation of e.s.d.'s in distances, angles and torsion angles; correlations between e.s.d.'s in cell parameters are only used when they are defined by crystal symmetry. An approximate (isotropic) treatment of cell e.s.d.'s is used for estimating e.s.d.'s involving l.s. planes.

### Fractional atomic coordinates and isotropic or equivalent isotropic displacement parameters (Å^2^)

**Table:**

	*x*	*y*	*z*	*U*_iso_*/*U*_eq_	
C6	0.3530 (9)	0.3279 (4)	0.4556 (7)	0.0389 (18)	
C5	0.3479 (11)	0.2939 (5)	0.5784 (8)	0.053 (2)	
H1A	0.4329	0.3102	0.6522	0.064*	
H1B	0.2637	0.314	0.5908	0.064*	
C4	0.4801 (10)	0.2932 (5)	0.4347 (9)	0.050 (2)	
H2A	0.4849	0.3154	0.3563	0.06*	
H2B	0.568	0.3073	0.5072	0.06*	
C7	0.3688 (11)	0.4178 (5)	0.4674 (9)	0.060 (3)	
H5A	0.4549	0.431	0.5408	0.091*	
H5B	0.2867	0.4399	0.479	0.091*	
H5C	0.3747	0.4389	0.3897	0.091*	
O1	0.1009 (7)	0.0872 (4)	0.5202 (7)	0.0698 (19)	
C1	0.1039 (9)	0.0605 (5)	0.4219 (9)	0.045 (2)	
C2	−0.0248 (11)	0.0383 (7)	0.3093 (10)	0.074 (3)	
H9A	−0.0107	−0.0129	0.279	0.111*	
H9B	−0.0418	0.0763	0.2412	0.111*	
H9C	−0.1064	0.0366	0.3338	0.111*	
I	0.43362 (5)	−0.00049 (3)	0.64352 (4)	0.03779 (16)	
Rh	0.29476 (7)	0.04485 (4)	0.40073 (6)	0.03779 (16)	
P	0.3320 (2)	0.17126 (11)	0.43763 (19)	0.0342 (4)	
O2	0.2452 (6)	−0.0699 (3)	0.3412 (5)	0.0382 (12)	
N1	0.1775 (7)	−0.0076 (4)	0.1406 (6)	0.0433 (16)	
O3	0.2017 (6)	0.0583 (3)	0.2034 (5)	0.0413 (13)	
N2	0.1995 (7)	−0.0702 (3)	0.2114 (6)	0.0366 (14)	
C11	0.1793 (8)	−0.1465 (4)	0.1476 (7)	0.0355 (17)	
O6	0.4702 (6)	0.2079 (3)	0.4228 (6)	0.0495 (14)	
O4	0.2045 (6)	0.2201 (3)	0.3337 (5)	0.0511 (15)	
O5	0.3416 (7)	0.2073 (3)	0.5719 (5)	0.0535 (16)	
C15	0.2426 (11)	−0.2819 (5)	0.1644 (10)	0.061 (3)	
H010	0.292	−0.3249	0.2131	0.073*	
C16	0.2521 (10)	−0.2091 (5)	0.2224 (8)	0.050 (2)	
H011	0.3069	−0.2026	0.3107	0.061*	
C13	0.0867 (11)	−0.2264 (6)	−0.0368 (10)	0.068 (3)	
H012	0.0298	−0.2321	−0.1248	0.082*	
C12	0.0962 (10)	−0.1545 (5)	0.0197 (8)	0.058 (2)	
H013	0.0463	−0.1114	−0.0288	0.07*	
C14	0.1587 (11)	−0.2907 (6)	0.0322 (10)	0.065 (3)	
H014	0.1518	−0.3393	−0.0083	0.078*	
C3	0.2178 (10)	0.3068 (4)	0.3415 (9)	0.051 (2)	
H01A	0.1346	0.3297	0.3522	0.061*	
H01B	0.2222	0.3275	0.262	0.061*	

### Atomic displacement parameters (Å^2^)

**Table:**

	*U*^11^	*U*^22^	*U*^33^	*U*^12^	*U*^13^	*U*^23^
C6	0.059 (5)	0.025 (4)	0.037 (4)	0.005 (4)	0.022 (4)	0.005 (3)
C5	0.086 (7)	0.039 (5)	0.042 (5)	0.001 (4)	0.033 (5)	−0.008 (4)
C4	0.064 (6)	0.030 (4)	0.064 (6)	−0.013 (4)	0.034 (5)	−0.010 (4)
C7	0.097 (8)	0.035 (5)	0.056 (6)	0.003 (5)	0.036 (5)	0.007 (4)
O1	0.070 (5)	0.070 (5)	0.085 (5)	0.003 (4)	0.047 (4)	−0.006 (4)
C1	0.050 (5)	0.040 (5)	0.053 (5)	0.010 (4)	0.027 (4)	0.004 (4)
C2	0.060 (7)	0.091 (8)	0.076 (7)	0.000 (6)	0.032 (6)	0.008 (6)
I	0.0466 (3)	0.0360 (2)	0.0325 (2)	0.00276 (18)	0.01725 (17)	0.00362 (16)
Rh	0.0466 (3)	0.0360 (2)	0.0325 (2)	0.00276 (18)	0.01725 (17)	0.00362 (16)
P	0.0421 (11)	0.0291 (10)	0.0324 (10)	0.0012 (8)	0.0155 (8)	0.0015 (8)
O2	0.058 (3)	0.024 (2)	0.035 (3)	−0.008 (2)	0.021 (3)	−0.002 (2)
N1	0.043 (4)	0.051 (4)	0.029 (3)	0.001 (3)	0.007 (3)	0.005 (3)
O3	0.054 (3)	0.024 (3)	0.044 (3)	0.001 (2)	0.017 (3)	0.007 (2)
N2	0.046 (4)	0.028 (3)	0.035 (3)	−0.003 (3)	0.015 (3)	0.001 (3)
C11	0.040 (4)	0.030 (4)	0.038 (4)	−0.004 (3)	0.017 (3)	−0.006 (3)
O6	0.051 (3)	0.032 (3)	0.076 (4)	−0.004 (3)	0.036 (3)	−0.007 (3)
O4	0.056 (4)	0.030 (3)	0.052 (3)	0.009 (3)	0.004 (3)	0.002 (3)
O5	0.100 (5)	0.032 (3)	0.038 (3)	−0.001 (3)	0.038 (3)	0.000 (2)
C15	0.078 (7)	0.039 (5)	0.064 (6)	0.004 (5)	0.025 (5)	−0.002 (4)
C16	0.067 (6)	0.038 (4)	0.041 (5)	0.002 (4)	0.016 (4)	−0.006 (4)
C13	0.071 (7)	0.066 (7)	0.053 (6)	−0.016 (5)	0.008 (5)	−0.022 (5)
C12	0.062 (6)	0.055 (6)	0.041 (5)	0.002 (5)	0.001 (4)	−0.005 (4)
C14	0.067 (7)	0.053 (6)	0.079 (7)	−0.021 (5)	0.033 (6)	−0.030 (5)
C3	0.072 (6)	0.026 (4)	0.054 (5)	0.010 (4)	0.022 (5)	0.003 (4)

### Geometric parameters (Å, °)

**Table:**

C6—C5	1.504 (10)	Rh—P	2.186 (2)
C6—C4	1.504 (11)	Rh—I^i^	3.0511 (9)
C6—C3	1.502 (12)	P—O6	1.588 (6)
C6—C7	1.533 (10)	P—O5	1.585 (5)
C5—O5	1.468 (9)	P—O4	1.589 (5)
C5—H1A	0.97	O2—N2	1.339 (7)
C5—H1B	0.97	N1—O3	1.290 (8)
C4—O6	1.451 (8)	N1—N2	1.289 (8)
C4—H2A	0.97	N2—C11	1.451 (9)
C4—H2B	0.97	C11—C12	1.352 (10)
C7—H5A	0.96	C11—C16	1.374 (11)
C7—H5B	0.96	O4—C3	1.474 (9)
C7—H5C	0.96	C15—C16	1.379 (11)
O1—C1	1.196 (10)	C15—C14	1.394 (13)
C1—C2	1.461 (13)	C15—H010	0.93
C1—Rh	2.040 (8)	C16—H011	0.93
C2—H9A	0.96	C13—C14	1.368 (14)
C2—H9B	0.96	C13—C12	1.358 (12)
C2—H9C	0.96	C13—H012	0.93
I—Rh	2.6351 (8)	C12—H013	0.93
I—Rh^i^	3.0511 (9)	C14—H014	0.93
Rh—O3	2.044 (5)	C3—H01A	0.97
Rh—O2	2.052 (5)	C3—H01B	0.97
			
C5—C6—C4	108.7 (7)	C1—Rh—I^i^	172.7 (2)
C5—C6—C3	110.0 (7)	O3—Rh—I^i^	85.36 (16)
C4—C6—C3	108.7 (7)	O2—Rh—I^i^	80.51 (15)
C5—C6—C7	110.0 (6)	P—Rh—I^i^	99.96 (6)
C4—C6—C7	109.6 (7)	I—Rh—I^i^	86.70 (2)
C3—C6—C7	109.8 (6)	O6—P—O5	102.2 (3)
O5—C5—C6	110.7 (6)	O6—P—O4	102.2 (3)
O5—C5—H1A	109.5	O5—P—O4	103.0 (3)
C6—C5—H1A	109.5	O6—P—Rh	117.2 (2)
O5—C5—H1B	109.5	O5—P—Rh	120.0 (2)
C6—C5—H1B	109.5	O4—P—Rh	110.0 (2)
H1A—C5—H1B	108.1	N2—O2—Rh	107.0 (4)
O6—C4—C6	111.8 (6)	O3—N1—N2	115.4 (6)
O6—C4—H2A	109.3	N1—O3—Rh	113.5 (4)
C6—C4—H2A	109.3	N1—N2—O2	124.3 (6)
O6—C4—H2B	109.3	N1—N2—C11	118.3 (6)
C6—C4—H2B	109.3	O2—N2—C11	117.3 (6)
H2A—C4—H2B	107.9	C12—C11—C16	122.0 (7)
C6—C7—H5A	109.5	C12—C11—N2	121.2 (7)
C6—C7—H5B	109.5	C16—C11—N2	116.7 (7)
H5A—C7—H5B	109.5	C4—O6—P	114.3 (5)
C6—C7—H5C	109.5	C3—O4—P	116.6 (5)
H5A—C7—H5C	109.5	C5—O5—P	114.7 (4)
H5B—C7—H5C	109.5	C16—C15—C14	119.7 (9)
O1—C1—C2	123.8 (9)	C16—C15—H010	120.2
O1—C1—Rh	120.9 (7)	C14—C15—H010	120.2
C2—C1—Rh	115.3 (7)	C11—C16—C15	118.8 (8)
C1—C2—H9A	109.5	C11—C16—H011	120.6
C1—C2—H9B	109.5	C15—C16—H011	120.6
H9A—C2—H9B	109.5	C14—C13—C12	121.8 (9)
C1—C2—H9C	109.5	C14—C13—H012	119.1
H9A—C2—H9C	109.5	C12—C13—H012	119.1
H9B—C2—H9C	109.5	C11—C12—C13	118.9 (9)
Rh—I—Rh^i^	93.30 (2)	C11—C12—H013	120.6
C1—Rh—O3	92.9 (3)	C13—C12—H013	120.6
C1—Rh—O2	92.2 (3)	C13—C14—C15	118.8 (8)
O3—Rh—O2	78.74 (19)	C13—C14—H014	120.6
C1—Rh—P	87.2 (2)	C15—C14—H014	120.6
O3—Rh—P	93.85 (14)	O4—C3—C6	108.6 (6)
O2—Rh—P	172.54 (15)	O4—C3—H01A	110
C1—Rh—I	93.9 (2)	C6—C3—H01A	110
O3—Rh—I	168.12 (14)	O4—C3—H01B	110
O2—Rh—I	91.25 (14)	C6—C3—H01B	110
P—Rh—I	96.21 (6)	H01A—C3—H01B	108.4

Symmetry codes: (i) −*x*+1, −*y*, −*z*+1.
